# Dedifferentiation of Primary Hepatocytes is Accompanied with Reorganization of Lipid Metabolism Indicated by Altered Molecular Lipid and miRNA Profiles

**DOI:** 10.3390/ijms20122910

**Published:** 2019-06-14

**Authors:** Mostafa Kiamehr, Laura Heiskanen, Thomas Laufer, Aneta Düsterloh, Mustafa Kahraman, Reijo Käkelä, Reijo Laaksonen, Katriina Aalto-Setälä

**Affiliations:** 1 BioMediTech, Faculty of Medicine and Health Technology, Tampere University, 33520 Tampere, Finland; reijo.laaksonen@zora.fi (R.L.); katriina.aalto-setala@tuni.fi (K.A.-S.); 2 Zora Biosciences, 02150 Espoo, Finland; laura.heiskanen@thermofisher.com; 3 Hummingbird Diagnostics GmbH, 69120 Heidelberg, Germany; tlaufer@hb-dx.com (T.L.); aduesterloh@hb-dx.com (A.D.); mkahraman@hb-dx.com (M.K.); 4 Department of Human Genetics, Saarland University, 66421 Homburg, Germany; 5 Clinical Bioinformatics, Saarland University, 66123 Saarbrücken, Germany; 6 Helsinki University Lipidomics Unit, Helsinki Institute for Life Science (HiLIFE) and Molecular and Integrative Biosciences Research Programme, University of Helsinki, FI-00014 Helsinki, Finland; reijo.kakela@helsinki.fi; 7 Heart Hospital, Tampere University Hospital, 33520 Tampere, Finland

**Keywords:** primary human hepatocytes (PHHs), dedifferentiation, lipidomics, mass spectrometry, sphingolipids (SLs), phospholipids (PLs), saturated fatty acids (SFAs), monounsaturated fatty acids (MUFAs), polyunsaturated fatty acids (PUFAs), microRNAs (miRNAs)

## Abstract

Aim: Primary human hepatocytes (PHHs) undergo dedifferentiation upon the two-dimensional (2D) culture, which particularly hinders their utility in long-term in vitro studies. Lipids, as a major class of biomolecules, play crucial roles in cellular energy storage, structure, and signaling. Here, for the first time, we mapped the alterations in the lipid profile of the dedifferentiating PHHs and studied the possible role of lipids in the loss of the phenotype of PHHs. Simultaneously, differentially expressed miRNAs associated with changes in the lipids and fatty acids (FAs) of the dedifferentiating PHHs were investigated. Methods: PHHs were cultured in monolayer and their phenotype was monitored morphologically, genetically, and biochemically for five days. The lipid and miRNA profile of the PHHs were analyzed by mass spectrometry and Agilent microarray, respectively. In addition, 24 key genes involved in the metabolism of lipids and FAs were investigated by qPCR. Results: The typical morphology of PHHs was lost from day 3 onward. Additionally, *ALB* and *CYP* genes were downregulated in the cultured PHHs. Lipidomics revealed a clear increase in the saturated fatty acids (SFA) and monounsaturated fatty acids (MUFA) containing lipids, but a decrease in the polyunsaturated fatty acids (PUFA) containing lipids during the dedifferentiation of PHHs. In line with this, *FASN*, *SCD*, *ELOVL1*, *ELOVL3*, and *ELOVL7* were upregulated but *ELOVL2* was downregulated in the dedifferentiated PHHs. Furthermore, differentially expressed miRNAs were identified, and the constantly upregulated miR-27a and miR-21, and downregulated miR-30 may have regulated the synthesis, accumulation and secretion of PHH lipids during the dedifferentiation. Conclusion: Our results showed major alterations in the molecular lipid species profiles, lipid-metabolizing enzyme expression as wells as miRNA profiles of the PHHs during their prolonged culture, which in concert could play important roles in the PHHs’ loss of phenotype. These findings promote the understanding from the dedifferentiation process and could help in developing optimal culture conditions, which better meet the needs of the PHHs and support their original phenotype.

## 1. Introduction

Primary human hepatocytes (PHHs) are commonly used as the “gold standard” model system to study liver physiology and disease, drug-induced liver injury, active drug transport mechanisms, and drug-drug interactions [1,2,3]. In addition, hepatocytes handle many crucial metabolic functions of the liver including the synthesis of fatty acids (FAs), cholesterol, cholesteryl esters, triacylglycerols (TAGs), and phospholipids (PLs), and utilizing these lipids together with apolipoproteins produce lipoprotein particles [4]. PHHs are sensitive to hepatitis C virus infection [5] and are able to express cytochrome P450 enzymes and drug transporters at significantly higher levels than most hepatoma cell lines such as HepG2 and Huh7 [6]. Since the availability of the PHHs is limited, scientists have tried to utilize other sources such as rodent primary hepatocytes or stem cell-derived hepatic cells as in vitro cell models. However, none of the so far developed hepatic models are yet as functional and relevant as PHHs in mimicking the complex physiology of the liver.

Upon liver injury, hepatic cells are able to rapidly proliferate and regenerate even a large area of the damaged organ [7]. Despite this extraordinary and unique feature, when hepatocytes are isolated and cultured in vitro, they are unable to expand and progressively lose their unique liver-specific functions [8,9], which typically limits their usability to a few days. This feature is known as “dedifferentiation” and significantly hampers the application of the PHHs particularly in long-term toxicity and xenobiotic biotransformation studies. It is known that the PHHs’ loss of phenotype is primarily a consequence of fundamental gene expression changes and diminished liver-enriched transcription factors triggered by the stress during isolation, disruption of the normal tissue architecture [8,10]. On the other hand, the current culture systems are not able to restore these phenotypical changes due to the lack of critical survival factors essential for liver-specific gene expression [8]. 3D culture of PHHs alone or together with microfluidic systems and in coculture with other cell types such as mesenchymal stem cells have been shown to improve the PHHs’ life span and functionality [11,12,13,14,15]. However, the conventional 2D culture of PHHs is still the most common approach due to its simplicity and low cost particularly for high-throughput studies. Although, in this culture system, PHHs undergo progressive deterioration of their in vivo-like morphological and functional phenotype [16].

The “-omics” approaches have been broadly utilized to map the changes in the transcriptome and proteome of the dedifferentiating hepatocytes [17,18,19,20,21]. It has been shown that proteins responsible for cytoskeletal remodelling as well as carbohydrate, amino acid and lipid metabolism networks are differentially expressed in dedifferentiating hepatocytes [21]. In addition, a recent study showed that energy production is decreased during the dedifferentiation through changes in the expression of mitochondrial-associated proteins, particularly those involved in FA and lipid metabolism [18]. Furthermore, a transcriptomic study on noncoding RNAs showed that FA metabolism is among the most affected pathways during dedifferentiation [22]. Despite the apparent importance of the lipids in the process of dedifferentiation, surprisingly, no study has been conducted yet on mapping the alterations in the lipid molecular species of the dedifferentiating hepatocytes. This has been partially due to the technical challenges, but the field of lipidomics is now well advanced and the approach using mass spectrometry can be utilized to better understand the role of molecular lipids in various physiological states including dedifferentiation. Such detailed analysis is required since mammalian cells produce thousands of lipids with various organelle-specific structural and functional roles. In addition, cells synthesize hundreds of proteins to control the lipid metabolism and their trafficking and secretion [23]. Therefore, an in-depth portrait of the temporal changes in the lipid profile and the associated genes and microRNAs (miRNAs) of the dedifferentiating hepatocytes is required to understand the complex protein-lipid interplay during the dedifferentiation process. With this new knowledge, we can optimize culture conditions and thereby improve both the phenotype and life span of the cultured PHHs.

Here, for the first time, we studied the alterations in the lipidome of the dedifferentiating primary human hepatocytes. To allow unrestricted dedifferentiation, PHHs were cultured in confluent 2D monolayer and their morphology, liver-specific gene expression, and function were monitored during the five days in culture. Most importantly, the lipidome changes in PHHs together with the alterations in the enzymes metabolizing FAs and lipids were studied during the dedifferentiation process. As miRNAs have been shown to be one important driver of hepatic dedifferentiation [22], alongside lipidomic studies, the temporal changes in the miRNA profile of the dedifferentiating PHHs were also investigated.

## 2. Results

### 2.1. The Morphology, Gene Expression, and Functionality of PHHs

**Figure 1:**
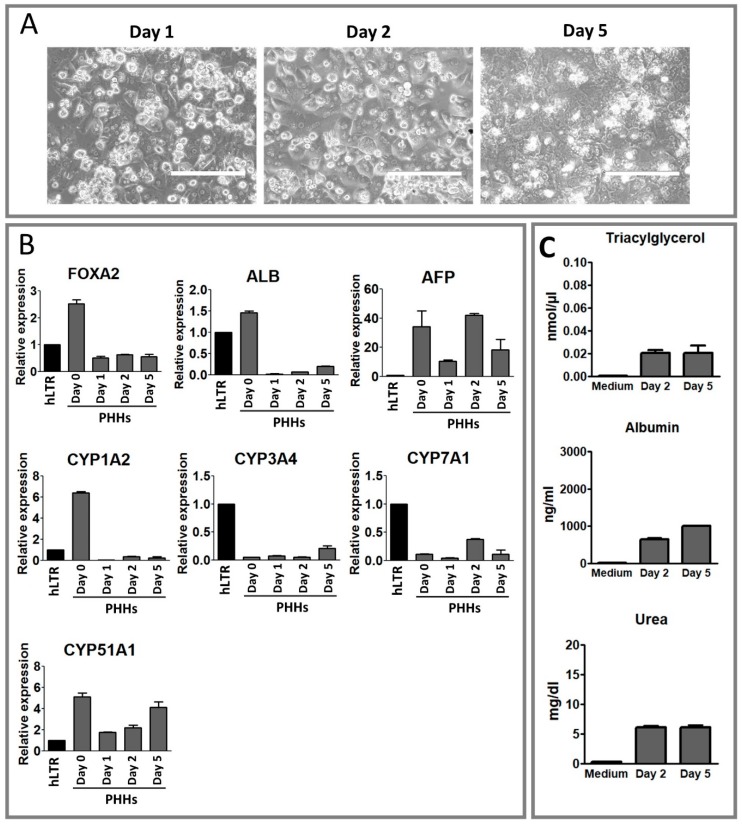
Evaluation of the morphology and functionality of primary human hepatocytes (PHHs) during the course of dedifferentiation. (**A**) Phase contrast images showing the morphology of the PHHs at days 1, 2, and 5 of the culture. The scale bar represents 200 µm. (**B**) qPCR analysis of *FOXA2*, *ALB*, *AFP*, *CYP1A2*, *CYP3A4*, *CYP7A1* and *CYP51A1* genes at days 0, 1, 2, and 5. The gene expression data for *FOXA2*, *ALB*, and *AFP* was normalized to housekeeping gene, *GAPDH*, and CYP genes were normalized to both *GAPDH* and *B2M*. The values are presented relative to the human liver total RNA (hLTR) sample. Each sample was run in technical triplicate and bars represent mean ± SD of three biological replicates. (**C**) Biochemical analysis of the conditioned media of the PHHs for secreted triacylglycerol (TAG), albumin, and urea at day 2 and 5 of the culture. Values are presented per well, per 24 h. Bars represent mean ± SD of at least three biological replicates.

PHHs remained viable and confluent throughout the five days in the culture. However, they started to lose their characteristic hepatocyte morphology from day 3 onward and at day 5, cells had completely lost their typical polygonal structure and exhibited a flattened appearance with weakly defined cell-to-cell borders (Figure 1A) which implied that the PHHs were dedifferentiating. 

Using qPCR, the expression of three liver-specific genes was assessed in the dedifferentiating PHHs (Figure 1B). *FOXA2* expression was downregulated by 5-fold at day 1 compared to day 0 and its expression remained low until day 5. Similar to *FOXA2*, the expression of *ALB* was dramatically downregulated by 58-fold at day 1 compared to day 0 and remained low afterwards. The expression of *AFP* fluctuated in the cultured PHHs and, depending on the time point, its level was 10- to 40-fold higher than that in our control sample, hLTR.

In order to assess the functionality of the PHHs at the gene level, the expression of four of the CYP isoforms known to be important in drug metabolism, lipid homeostasis, and cholesterol biosynthesis [24,25,26] was evaluated in the dedifferentiating PHHs (Figure 1B). *CYP1A2* was downregulated by about 120-fold after the PHHs were cultured for one day. Similarly, *CYP7A1* and *CYP51A1* were both downregulated by approximately 2- and 3-fold during the first day of culture. The expression of *CYP3A4*, was downregulated already before the culture since it’s expression levels at day 0 was 18-fold lower than that in the reference hLTR sample. Interestingly, the expression of all four CYP genes was slightly recovered either at day 2 or day 5 of the cell culture.

The functionality of the PHHs was assessed also biochemically by measuring the secreted TAG, albumin, and urea in the medium of the cultured PHHs at day 2 and 5. No changes were observed in the ability of the PHHs to secrete TAG and urea between day 2 and day 5 (Figure 1C). Interestingly however, the amount of albumin secreted by PHHs at day 5 had increased by 55% compared to its amount at day 2, which correlated well with our observation from the expression of *ALB* at the gene level.

### 2.2. Alterations in the Lipid Profile of the PHHs

**Figure 2:**
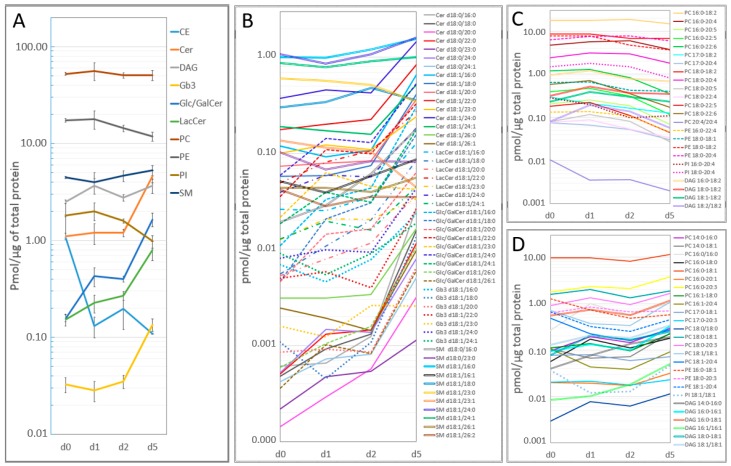
Alterations in the lipid profile of the PHHs during the dedifferentiation. Lines describe the detected concentration (pmol/µg total protein) of (**A**) different lipid classes, (**B**) molecular sphingolipid (SL) species, and (**C**,**D**) molecular phospholipid (PL) and diacylglycerol (DAG) species detected in PHHs at days 0, 1, 2, and 5; ’d’ represents days in culture. Panel C shows the PL molecular species that decreased, and panel D the species that increased during the dedifferentiation. Bars in A represent mean ± SD of three biological replicates. The detailed values of lipidomics is provided in the Table S2. CE = cholesteryl ester, Cer = ceramide, DAG = diacylglycerol, Gb3 = globotriaosylceramide, Glc/GalCer = glucosyl/galactosylceramide, LacCer = lactosylceramide, PC = phosphatidylcholine, PE = phosphatidylethanolamine, PI = phosphatidylinositol, SM = sphingomyelin.

The lipid profile in the dedifferentiating PHHs was studied by mass spectrometry and a total of 139 molecular species spanning 19 cholesteryl esters (CEs), 16 ceramides, 10 diacylglycerols (DAGs), 7 globotriaosylceramides (Gb3s), 9 glucosyl/galactosylceramides (Glc/GalCers), 7 lactosylceramides (LacCers), 2 lyso-phosphatidylcholines (LPCs), 7 lyso-phosphatidylethanolamines (LPEs), 1 lyso-phosphatidyl glycerol (LPG), 3 lyso-phosphatidylinositols (LPIs), 1 lyso-phosphatidylserine (LPS), 30 phosphatidylcholines (PCs), 7 phosphatidylethanolamines (PEs), 3 phosphatidylinositols (PIs), and 17 sphingomyelins (SMs) were detected in the cultured PHHs at days 0, 1, 2, and 5 (Table S2). Our data showed that the levels of sphingolipids (SLs) increase, but the levels of phospholipids (PLs) decrease in the dedifferentiating PHHs (Figure 2A). In fact, the total concentration of ceramide, LacCer, Glc/GalCer, and Gb3 increased in PHHs by 4.8-, 5.3-, 10.3-, and 4-fold, respectively from day 0 to day 5. On the other hand, the total concentration of PE and PI decreased by about 1.5- and 2-fold respectively, from day 0 to day 5. The total PC remained relatively constant during the culture of the PHHs. The total CE content was reduced in PHHs-d1 by almost 10-fold compared to PHHs-d0 and its concentration remained low during the rest of the culture. The total amount of the DAG fluctuated in PHHs.

Monitoring the individual molecular species in PHHs during the culture period demonstrated that the concentration of almost all molecular SL species was increasing over time, particularly between day 2 and day 5 (Figure 2B). The only exceptions were Glc/GalCer d18:1/23:0, SM d18:0/23:0, SM d18:1/23:0, SM d18:1/23:1, and SM d18:1/26:2. Interestingly, the increase was more pronounced in SLs containing long-chain FAs (LCFAs, C16-22) compared to those SLs containing very-long-chain FAs (VLCFAs, C22-26).

The concentration of PL molecular species also showed temporal changes, and based on the alterations in their concentrations between day 2 and day 5, the PLs could be divided into two groups (Figure 2C,D). In the first group, the species were mainly composed of polyunsaturated FAs (PUFAs, specially 18:2) or highly unsaturated FAs (HUFAs, with 4–6 double bonds) coupled with a saturated FA (SFA). The concentrations in this group were mainly decreasing from day 2 to day 5 (Figure 2C). The second group largely consisted of SFAs and/or monounsaturated FAs (MUFAs), and the PUFA chains found in this group of lipids were restricted to the 20:3 and 20:4 chains. The concentrations in this group, in contrast to the first group, were mainly increasing between day 2 and day 5 (Figure 2D).

### 2.3. Alterations in the Lipid Metabolism-Related Genes

**Figure 3:**
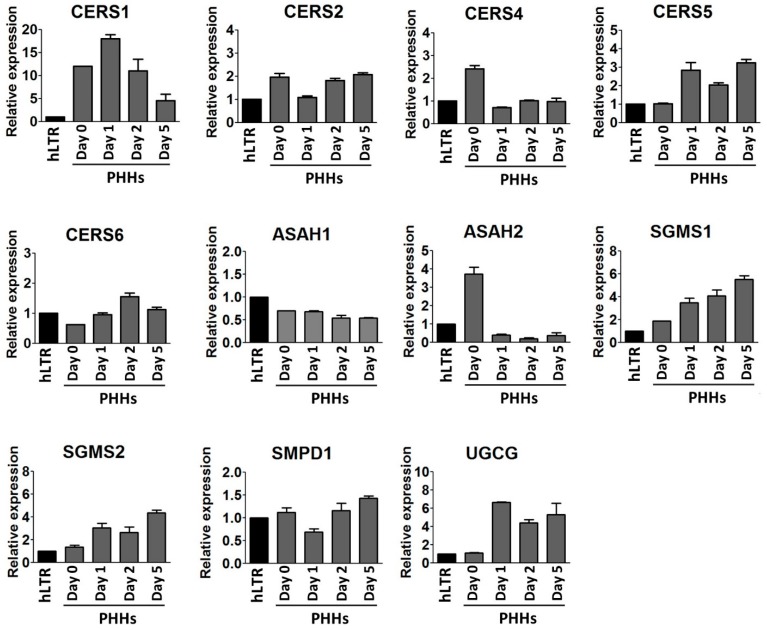
qPCR analysis of key genes involved in the metabolism of sphingolipids (SLs) studied in PHHs during their five days in the culture. The expression of *CERS1*, *CERS2*, *CERS4*, *CERS5*, *CERS6*, *ASAH1*, *ASAH2*, *SGMS1*, *SGMS2*, *SMPD1*, and *UGCG* genes in PHHs at time points day 0, 1, 2, and 5. The expression of each gene was normalized to both *GAPDH* and *B2M* as endogenous controls. The values are presented relative to the hLTR sample. Each sample was run in technical triplicate and bars represent mean ± SD of three biological replicates.

**Figure 4:**
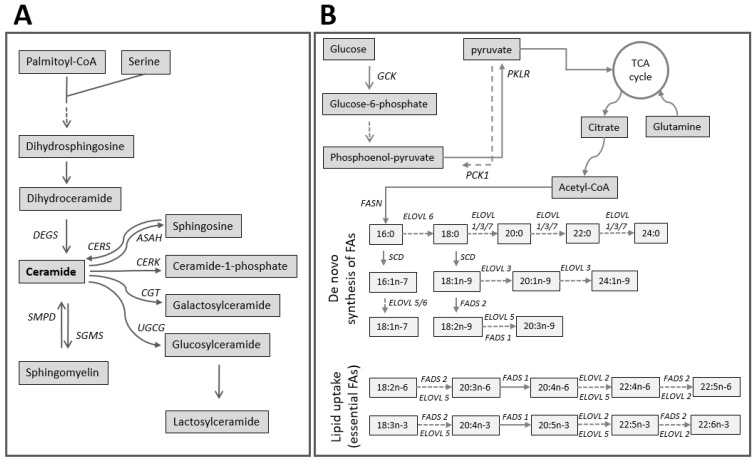
(**A**) Demonstrates a simplified pathway of sphingolipids (SLs) together with the responsible key genes involved in regulating the pathway. (**B**) Demonstrates a simplified pathways of de-novo synthesis of fatty acids (FAs) as well as imported essential polyunsaturated FAs (PUFAs) together with responsible genes involved in regulating the pathway (the figure was adapted from Kiamehr et al. [27]).

Several genes related to the synthesis of SLs were evaluated by qPCR in the dedifferentiating PHHs (Figure 3). Ceramide synthesis is orchestrated by a family of six CerSs encoded by *CERS1*-*CERS6* (Figure 4A). *CERS1* and *CERS5* were upregulated by 1.5- and 3-fold, respectively, in PHHs-d1 compared to PHHs-d0. *CERS1*, however, was gradually downregulated while *CERS5* remained upregulated until the end of the culture. In contrast to *CERS1* and *CERS5*, *CERS4* was downregulated by 3-fold in PHHs-d1 and remained relatively constant afterwards. The expression of *CERS6* was upregulated by 2-fold in PHHs-d2 compared to PHHs-d0. *CERS2* was expressed relatively constant throughout the entire culture.

Ceramides are degraded to sphingosine and free FAs by ceramidases encoded by distinct genes like *ASAH1* and *ASAH2* (Figure 4A). The expression of *ASAH1* remained constant throughout the culture, while *ASAH2* was downregulated in PHHs by 9-fold after one day in the culture. The expression of *ASAH2* remained low until day 5. On the other hand, the expression of *SGMS1* and *SGMS2* involved in the production of SM from ceramides were constantly upregulating and in PHHs-d5, both *SGMS1* and *SGMS2* were expressed approximately 3-fold higher compared to their levels in PHHs-d0. SM is hydrolyzed by isoforms of sphingomyelinase (SMase) which produce phosphorylcholine and the intracellular effector ceramide. *SMPD1* gene encodes a lysosomal acid sphingomyelinase and its expression remained constant during the cell culture of the PHHs.

The UDP-glucose:ceramide glucosyltransferase (*UGCG*) gene encodes the enzyme which catalyzes the first glycosylation step in glycosphingolipid (GSL) biosynthesis. In the cultured PHHs, *UGCG* was upregulated by 6-fold at day 1 compared to day 0 and remained upregulated during the rest of the culture.

### 2.4. Alterations in FA Metabolism-Related Genes

**Figure 5:**
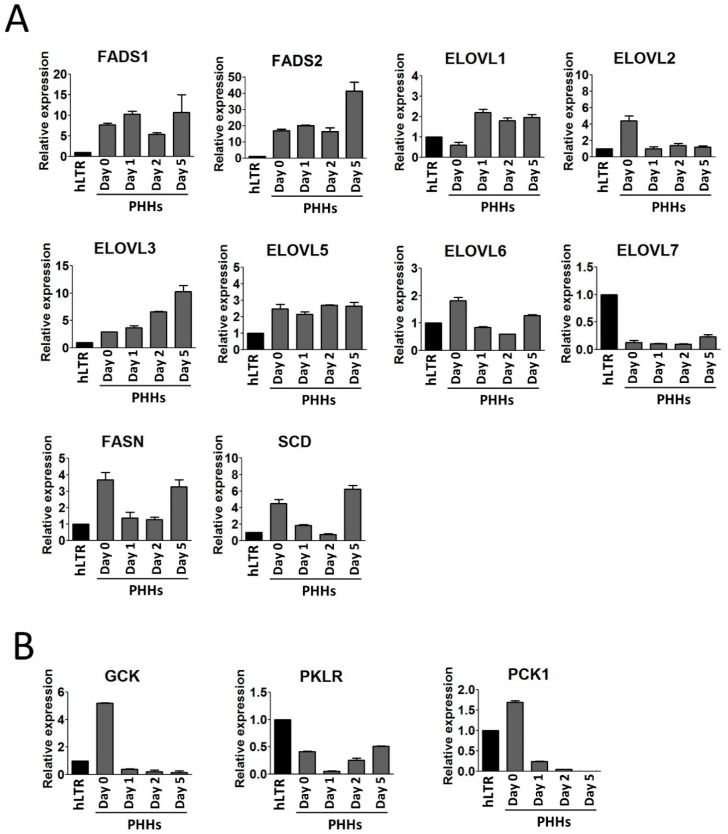
Real-time qPCR analysis of genes involved in the metabolism of fatty acids (FAs). (**A**) and glucose homeostasis (**B**) studied in dedifferentiating PHHs. The expression of (**A**) *FASN*, *SCD*, *FADS1*, *FADS2*, *ELOVL1*, *ELOVL2*, *ELOVL3*, *ELOVL5*, *ELOVL6*, and *ELOVL7* and (**B**) *GCK*, *PKLR*, and *PCK1* at time points day 0, 1, 2, and 5. The expression of each gene was normalized to both *GAPDH* and *B2M* as endogenous controls. The values are presented relative to the hLTR sample. Each sample was run in technical triplicate and bars represent mean ± SD of three biological replicates.

FA desaturase (FADS) inserts double bonds to pre-existing PUFA precursors (Figure 4B). In our study, *FADS1* remained unchanged and *FADS2* were upregulated by almost 3-folds at day 5. The genes related to the elongation of very- long-chain fatty acids (*ELOVL*) work in sequence with the desaturases and are essential for the metabolism of saturated or unsaturated LCFAs and VLCFAs (Figure 4B). *ELOVL1*, *ELOVL3*, and *ELOVL7* are involved in the elongation of saturated FAs while *ELOVL2* and *ELOVL5* are mainly involved in elongation of unsaturated FAs. We observed that *ELOVL1* was upregulated in PHHs-d1 by 4-fold compared to the expression level in PHHs-d0, and its expression remained upregulated until day 5. *ELOVL3* was constantly upregulated from day 0 to day 5. *ELOVL7* was expressed at low levels on days 0–2 but was upregulated by 2-fold at day 5 when compared to the level found on day 2. On the other hand, *ELOVL2*, was downregulated in PHHs-d1 by 3-fold compared to PHHs-d0 and remained low afterwards. Genes active in the beginning of the process of de novo synthesis of FAs such as FA synthase (*FASN*), Stearoyl-CoA desaturase (*SCD*), and *ELOVL6* showed the same pattern of expression and were downregulated in the beginning of the culture but were upregulated and recovered to their original expression towards the end of the culture (Figure 5A).

### 2.5. Alterations in the Genes Related to Glucose Homeostasis

Since the lipid metabolism in hepatocytes is highly affected by glucose homeostasis (Figure 4B), we studied the three key genes involved in glycolysis (*GCK* (encoding glucokiase) and *PKLR* (encoding liver-type pyruvate kinase)) and gluconeogenesis (*PCK1* (encoding phosphoenolpyruvate carboxykinase, thus also known as *PEPCK*)) of the liver (Figure 5B). All three genes, *GCK*, *PKLR*, and *PCK1*, were downregulated in PHHs after one day in the culture. The expression of both *GCK* and *PCK1* was further downregulated in PHHs-d2 and PHHs-d5. *PKLR* however, was upregulated in PHHs-d2 and recovered its original level of expression at day 5 of the culture.

### 2.6. Alterations in the miRNA Profile of the PHHs

**Figure 6:**
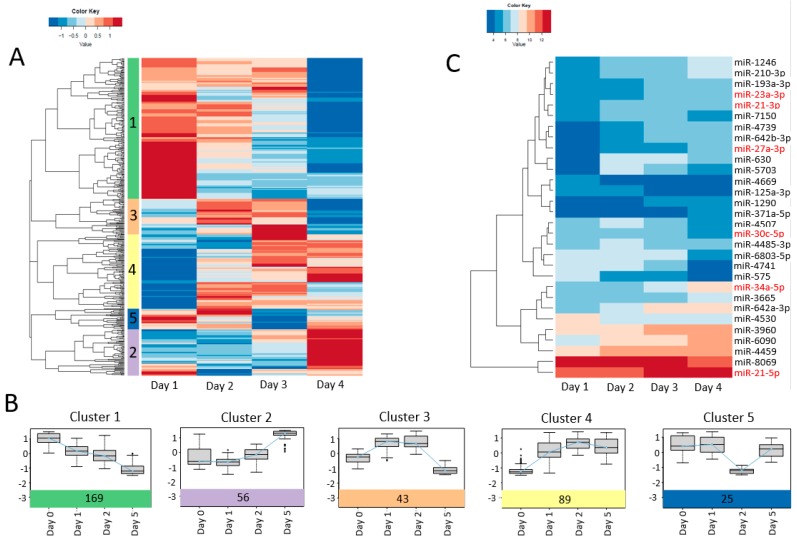
Influence of prolonged culture of PHHs on their miRNAs expression detected by microarrays. (**A**) Unsupervised hierarchical clustering of miRNAs using Eucledian distance and complete linkage in the samples. Columns indicate the days of the culture. The expressions of the miRNAs are row-scaled (by time point) and colour coded with blue indicating decreased and red indicating increased expression. miRNAs are clustered in five distinct groups according to their expression pattern marked with coloured boxes and numbers on the left side of the heatmap. The detailed list of miRNAs belonging to each cluster is provided in the Table S3. (**B**) To better illustrate the alterations of miRNAs presented in panel A, box-plots are prepared for each individual cluster at time points day 0, day1, day 2, and day 5. Each box-plot represents the average row-scaled expression of all miRNAs in that specific time point and the bars represent the mean ± SD of the collective miRNAs. Colours on the bottom of each plot is matching the colour of the clusters in the panel A. The numbers of miRNAs included in each cluster are shown in their respective coloured area. (**C**) Heatmap representing the expression changes of top 30 miRNAs in cultured PHHs on days 1, 2, and 5. Red font colour indicate the miRNAs already associated with the functionality and lipid metabolism of PHHs.

In total, 382 miRNAs were detected in the cultured PHHs at time points day 0, 1, 2, and 5 (Table S3). A heatmap was prepared from all 382 miRNAs detected and according to their expression pattern, miRNAs could be divided into five distinct clusters (Figure 6A,B). Our analysis showed that 23 miRNAs were upregulated and 22 miRNAs were downregulated in PHHs-d5 compared to those in PHHs-d0. The miR-34a, miR-27a, and miR-1246 were the most upregulated and miR-575, miR-4741, and miR-8069 were the most downregulated miRNAs in PHHs-d5. A separate heatmap was prepared from the 30 most differentially expressed miRNAs (Figure 6C). Several differentially expressed miRNAs (shown in red of Figure 6C) have been already associated with functionality or regulating the lipid metabolism of the cells (see Discussion Section).

## 3. Discussion

Lipid and FA synthesis pathways are dramatically affected during the process of dedifferentiation in cells [18,21,22]. However, the exact alterations and their possible role in the rapid dedifferentiation of the PHHs is not yet understood. Here, for the first time, we have performed a comprehensive lipidomic and lipid-related gene analysis on dedifferentiating PHHs alongside with miRNA analysis. We observed major alterations in both lipid and miRNA profiles of the PHHs during their prolonged 2D culture. Furthermore, de novo synthesis of SFAs and MUFAs was upregulated while PUFA synthesis seemed to be downregulated in the dedifferentiating PHHs, which was clearly mirrored in the increased levels of SLs and MUFA containing PLs and decreased levels of PUFA containing PLs.

PHHs remained viable during the culture, however, the liver-specific functions, as indicated by *ALB* and *CYP1A2* expression were deteriorating rapidly after plating. In addition, *CYP3A4* and *CYP7A1* were expressed already in PHHs-d0 considerably lower than those in freshly isolated liver samples (hLTR), indicating the diminished expression of those genes even before plating and possibly during the isolation and perfusion process. It is known that dedifferentiation can be triggered already during the isolation process, when organized and polarized cellular architecture of the liver is disrupted and ischemia-perfusion injury occurs. As a result, liver-enriched transcription factors and CYP enzymes become downregulated and hepatocytes rapidly deteriorate [8,10,28]. Interestingly, the downregulated *ALB* and *CYP* genes were slightly upregulated after 2 or 5 days in culture, which may imply the partial adaptation of the PHHs to the 2D culture environment [8,29]. Dedifferentiation is a complex, differentially controlled, and active process [18] and while the mRNA expression related to some enzymes become downregulated, the transcription of others might stay unchanged or even over-expressed during the prolonged culture of PHHs. However, it should be recalled that the fluctuations in mRNA levels only indicate the prevailing metabolic responses of the cells but the patterns do not necessarily correspond to the fluctuations in the steady-state cellular contents of the functional proteins or enzymes.

The lipidomic analysis showed that the concentration of almost all SL species increased in the dedifferentiating PHHs, and most increases were detected in Glc/GalCer species. SLs have been classically associated to cell permeability; however, their roles in intercellular communication and cell signaling are now well recognized. SLs are involved in the regulation of cell growth, differentiation, senescence, necrosis, proliferation, and apoptosis [30]. The metabolism of SLs is a complex process consisting of multiple metabolic pathways with ceramide acting as a central key molecule [31]. Ceramide can be further metabolized to SM or GlcCer, the first step in the metabolism of the glycosphingolipid (GSL) family. GSLs are involved in various cellular events such as signaling, trafficking, and cellular interactions [32]. To better understand the alterations in SLs, we further studied the key genes involved in the metabolism of SLs and we observed that several genes such as *SGMS1* and *UGCG*, involved in the synthesis of SLs, were upregulated and simultaneously, *ASAH2* involved in the degradation of SLs was downregulated in the dedifferentiated PHHs. These findings were in line with our lipidomics observations of elevated SL contents. In addition, we detected a temporal upregulation of *CERS1* (synthesizing C18 ceramides) and upregulation of *CERS5* (synthesizing C16 ceramides), but a downregulation of *CERS4* (synthesizing C18-20 ceramides) in the cultured PHHs. In agreement with this changes, the ceramide species with the 16:0 or 18:0 acyl chain showed the most increases during the process of dedifferentiation. Ceramide synthesis is orchestrated by a family of six CerSs (CerS1-CerS6), each of which produce ceramides with distinct FA chain lengths and degree of unsaturation [33]. The FA composition of a ceramide determines its biological function [33,34] and different ceramides are known to be involved in regulating the balance between cell death and survival [35]. The upregulation of *CERS1* and *CERS5* but downregulation of *CERS4* in dedifferentiating PHHs may be attributable to the activation of a pro-survival or anti-apoptotic pathway in PHHs. It is known that many of the earliest changes during the dedifferentiation are associated to cell survival or maintenance of homeostatic functions [18]. These alterations could act as a double-edged sword, on one hand preventing apoptosis, while on the other hand, playing a critical role in the loss of liver-specific functions. Therefore, we think that the alteration in the expression of CerSs could play an important role in both survival and dedifferentiation of the PHHs.

Our study showed that the concentration of SM species increased during the dedifferentiation and this increase was concurrent with upregulation of both *SGMS1* and *SGMS2* expression. SMS1 and SMS2 enzymes are, to current knowledge, the most responsible for SM de novo synthesis [36]. Approximately two thirds of cellular SM amount is located on the plasma membrane and about 65% of membrane SM is located in lipid rafts [37]. Lipid rafts are specialized microdomains of plasma membrane enriched with very-long-chain and saturated SLs, unlike the surrounding bulk membrane, which contains unsaturated PLs [38,39]. Therefore, our findings implicate that the organization of the cell membrane and dynamics of the lipid rafts are altered during the dedifferentiation, which would critically affect cell signaling, membrane trafficking, as well as membrane fluidity and permeability, ultimately resulting in compromised cellular functionality.

FAs are building block of lipids, therefore, we also studied the expression of key genes involved in the metabolism of FAs. A list of studied lipid and FA metabolism related genes and their function has been summarized in Table 1. We observed an upregulation in the de-novo synthesis of SFAs and MUFA synthesis pathways but a downregulation in the production of PUFAs in the dedifferentiating PHHs. In the cytoplasm, acetyl-CoA is carboxylated to malonyl-CoA, which is used as a precursor by FAS enzyme to synthesize palmitic acid (16:0 FA) [40]. The 16:0 is then elongated by ELOVL members in the ER to generate LCFAs (>16 carbon). The LCFAs are desaturated by *SCD* to produce MUFAs. These MUFAs are utilized by the cells for the production of e.g., PLs and TAGs [41]. *FASN*, *ELOVL6*, and *SCD* showed the same pattern of expression, suggesting that they regulated the de novo FA synthesis in concert during the prolonged culture of the PHHs and interestingly, this pattern of expression was mirrored in the levels of the PL species containing FA 18:1 (e.g., PC 18:1/18:1 and PI 18:1/18:1) (Figure 2D). *ELOVL1*, *ELOVL3*, *ELOVL6*, and *ELOVL7* have a putative role in the elongation of SFAs and MUFAs, and *ELOVL2* and *ELOVL5* are known as PUFA elongases [42]. *ELOVL1* and *ELOVL3* were upregulated during the dedifferentiation of the PHHs, confirming our observed increase in the levels of SFAs and MUFAs. On the other hand, *ELOVL2* became downregulated in the dedifferentiating PHHs, in line with our observations of reduced concentrations of PL species containing a very-long chain PUFA chain. The PHHs synthesize the very-long chain and highly unsaturated FAs from the PUFA precursors (FAs 18:2 or 18:3) which are taken up from the culture medium. Reduced levels of PUFAs, but increased levels of MUFAs in the dedifferentiated PHHs may imply that their ability to uptake the 18:2 precursor, available abundantly in their medium was altered due to membrane structure modifications. This could drive PHHs to compensate their PUFA deficiency by increasing the production of MUFAs [43].

FAs are a major source of energy, regulated by a variety of factors acting in concert to maintain the energy homeostasis within the cells. Glucose—a precursor for Acetyl-CoA—is one main regulator and its metabolism is controlled by various pathways including glycolysis and gluconeogenesis [51,52]. Downregulation of both *GCK* and *PCK1* in dedifferentiating PHHs points towards a decrease in glycolysis and gluconeogenesis respectively, resulting in the lower production of Acetyl-CoA, a precursor for synthesis of FAs. Despite this, we observed that de novo FA synthesis increased in dedifferentiated PHHs. This may imply that PHHs utilized Acetyl-CoA produced from other precursors than glucose such as glutamine [52] available in the culture medium. Nevertheless, the deterioiration of in vivo-like morphological and functional phenotype of PHHs is a very complex process, which likely involves also other regulatory factors than those investigated in the current study. For instance, the fundamental integration of hepatic glucose and ipid metabolism, which employs counteracting SREBP-1 and ChREBP signaling, could also be affected in the dedifferentiation process of PHHs [53].

Recently, miRNAs were shown to play important roles in the PHHs’ loss of phenotype [22,54]. In addition, emerging evidence shows that miRNAs are important regulators of lipoprotein formation and secretion, lipid synthesis, and FA oxidation [55]. Therefore, alongside lipidomics, we performed miRNA analysis on dedifferentiating PHHs to identify the miRNAs that possibly regulate the lipid changes during the dedifferentiation. We mainly focused on miRNAs with an established role in the regulation of lipid and FA metabolism or linked to the functionality of the cells and interestingly, we identified several miRNAs such as miR-34a, miR-23a, miR-27a (included in cluster 2), miR-21 (included in cluster 4), and miR-30c and miR-122 (included in cluster 1) among up- or downregulated miRNAs [55,56,57,58,59]. The miR-34a was the most upregulated miRNA and is among the most studied miRNAs with multiple known roles in the regulation of cell cycle, differentiation, migration, and apoptosis [55]. In addition, miR-34a is one of the regulators of HNF4α [58]. HNF4α positively regulates the expression of bile acid-synthesizing enzymes such as CYP7A1 [60]. Furthermore, miR-34a negatively regulates retinoid X receptor alpha (RXRα) resulting in decreased induction of CYP26 family and CYP3A4 [61]. Moreover, it has been shown that miR-34a targets Sirtuin 1 (SIRT1), which regulates a series of genes and proteins involved in lipid and glucose metabolism [62,63,64]. Another upregulated miRNA in the dedifferentiated PHHs was miR-23a, which is known to suppress gluconeogenesis through *PGC-1α* and *G6PC*, leading to decreased glucose production in the hepatic cells [65,66]. Therefore, the upregulation of miRNa-34a and/or miRNA-23a could be associated with the downregulation of *GCK*, *PCK1*, and CYP genes, and the loss of functionality of the dedifferentiated PHHs.

Similar to miR-34a, miR-27a is also shown to target RXRα. In addition, it can regulate ATP-binding cassette transporter A1 (ABCA1) [55,67]. Overexpression of miR-27a has been shown to accelerate adipolysis and repress lipid storage in the cells [55]. In addition, miR-27a inhibits the expression of FASN, SCD1, sterol regulatory element-binding proteins (SREBP-1 and SREBR-2), PPARα and PPARγ, APOA1, APOB100, and APOE3 [56,68]. Therefore, it has been suggested that miR-27 may regulate the metabolism of lipids by increasing lipid secretion and reducing lipid synthesis in the cells [55]. In our study, miR-27a was constantly upregulated in the dedifferentiating PHHs which occurred simultaneously with the downregulation of both *FASN* and *SCD* in PHHs-d1 and PHHs-d2, but not in PHHs-d5 where the expression of those genes was upregulated. This suggests that other miRNAs could be involved in the regulation of the *FASN* and *SCD* in PHHs. miR-21 (included in cluster 4) was another miRNA which showed constant upregulation in dedifferentiating PHHs. miR-21 has shown to target fatty acid-binding protein 7 (FABP7) and blocking C 18:0-induced intracellular lipid accumulation [69]. In addition, miR-21 directly targets PPARα and plays a role in development of steatosis by inhibition of PPARα-mediated FA uptake and lipid oxidation [59,70]. Together with liver X receptor (LXR) and insulin agonists, PPARα agonists can induce lipogenic gene expression such as *FASN* [71]. In addition, it has been recently reported that miR-30c directly targets *FASN*, resulting in reduced TAG accumulation in the cells [72]. In our study, miR-30c was downregulated in PHHs-d5 simultaneously with the upregulation of *FASN*. In another study the microsomal triglyceride transfer protein (MTP), important for lipoprotein formation, was found to be regulated by miR-30c [73]. Therefore, miR27a, miR21, and miR-30c could be working together to regulate the synthesis, accumulation, and secretion of lipids in PHHs during the process of dedifferentiation. Expectedly, miR-122 was also downregulated in dedifferentiated PHHs. miR-122 is not only regarded as a hepatic-specific marker but also is the first identified miRNA to regulate lipid metabolism [55]. miR-122 is also known to be essential for maintaining the hepatocellular phenotype [74,75]. Interestingly, the phenotypic and morphological loss of PHHs was concurrent with a steep reduction in the expression of miR-122 on day 5.

Taken together, this study provides novel findings on the possible roles of lipids, FAs, their corresponding metabolising enzymes, and the miRNAs in the process of dedifferentiation. This data may be applied to unravel the mechanisms behind the fast deterioration of the PHHs and developing culture conditions that would better support the phenotype of the PHHs in the culture for long-term studies. In addition, this knowledge could be utilized in the development of novel strategies for improving protocols for differentiation and maturation of stem cell-derived hepatocytes.

## 4. Method and Materials

### 4.1. Cell Culture

Cryopreserved PHHs (Cat. No. HMCPIS, Lot. HU8210, 51-year-old Caucasian male) were purchased from Gibco^®^ and were thawed and plated according to the manufacturer’s instructions. PHHs were cultured in monolayer on collagen-I-coated plates (CellAffix^TM^, AP Sciences Inc., Columbia, MD, USA), 2.5 × 10^5^ cells/cm^2^, and maintained in William’s E medium (A1217601, Gibco; without L-glutamine) supplemented with cocktail B (CM 4000, Gibco; Durham, NC, USA, including ITS (insulilne, transferine, selenium), GlutaMAX^TM^, BSA, and linoleic acid [18:2n-6] as the main FA constituent with 89 mol%, as detected by gas chromatography in the batch used for this study) and dexamethasone for up to 5 days. PHHs that were collected immediately after plating were considered as samples day 0. PHHs were also collected at days 1, 2, and 5.

### 4.2. Real-Time Quantitative PCR Analysis

RNA samples were collected (three biological replicate per time point) from PHHs at days 0, 1, 2, and 5 by RNeasy kit (Qiagen, Cat. No. 74106). cDNA was generated by High Capacity cDNA Reverse Transcription kit (Applied Biosystems, Ref. No. 4368814, Vilnius, Lithuania) according to the manufacturer’s instructions in the presence of RNase inhibitor. cDNA was multiplied either by Power SYBR Green PCR Master Mix (Applied Biosystems, Cat. No. 4367659, Warrington, UK) and gene specific primers (*FOXA2*, *AFP*, *ALB*) or Taqman Fast Advanced Master Mix (applied Biosystems, Ref. No. 4444557, Austin, TX, USA) and gene specific TaqMan probes (*CYP1A2*, *CYP3A4*, *CYP7A1*, *CYP51A1*, *CERS1*, *CERS2*, *CERS4*, *CERS5*, *CERS6*, *ASAH1*, *ASAH2*, *SGMS1*, *SGMS2*, *SMPD1*, *UGCG*, *FASN*, *FADS1*, *FADS2*, *SCD*, *ELOVL1*, *ELOVL2*, *ELOVL3*, *ELOVL5*, *ELOVL6*, *ELOVL7*, *GCK*, *PKLR*, and *PCK1*) using BioRad CFX384 Real Time PCR Detection System (Optic Module, Singapore). List of primers and TaqMan assays used in this study are presented in Table S1a,b. Values from *FOXA2*, *AFP*, and *ALB* were normalized to *GAPDH* and the rest to both *GAPDH* and *B2M* used as endogenous controls. Relative quantification was calculated by ∆∆CT method using Bio-Rad CFX Manager software, version 3.1 (Hercules, CA, USA). Fold change 2 was considered as a cut off value when considering a gene to be up- or downregulated.

First Choice^®^ Human Liver Total RNA (hLTR, Invitrogen^TM^, Cat. No. AM7960) was purchased from Ambion^®^ and was used as a reference sample.

### 4.3. Triacylglycerol, Albumin, and Urea Secretion

At days 2 and 5 of the cell culture, PHHs were evaluated for their functionality using biochemical assays (three to four biological replicates per time point). TAG, albumin, and urea content of the PHHs’ conditioned medium were determined, respectively, by triglyceride quantificaiton kit (BioVision Inc., Milpitas, CA, USA, Cat. No. K622-100), human albumin ELISA quantitation kit (Bethyl Laboratory, Montgomery, TX, USA), and QuantiChrom^TM^ urea assay kit (BioAssay Systems, Hayward, CA, USA) according to the manufacturers’ instructions. The results were then compared to the unconditioned William’s E medium.

### 4.4. Lipid Mass Spectrometry

Lipids (glycerolipids, SLs, GSLs and CEs) were extracted from PBS-resuspended PHHs on days 0, 1, 2, and 5 (three replicates per time point) by a modified Folch lipid extraction, using chloroform, methanol, and acetic acid for liquid-liquid extraction [76] employing a Hamilton Microlab Star system (Hamilton Robotic AB, Kista, Sweden). Details of the extraction procedure have been published before [77].

CE, DAG, and SM were analysed using the shotgun approach on a hybrid triple quadrupole/linear ion trap mass spectrometer (QTRAP 5500) with nanoflow ion source (NanoMate, Advion Biosciences Inc., Ithaca, NY, USA) as described by Heiskanen et al. [78]. Molecular species were analyzed in positive ion mode using lipid class-specific precursor ion or neutral loss scans [79,80]. Ceramide, Glc/GalCer, LacCer, Gb3, PC, PE, and PI were analysed using the targeted approach by ultra-high-pressure liquid chromatography-mass spectrometry (UHPLC-MS) [81]. An analytical Acquity BEH C18, 2.1 × 50 mm column with a particle size of 1.7 μm (Waters, Milford, MA, USA) was used with the mobile phases containing 10 mM ammonium acetate in water with 0.1% formic acid (solvent A), and 10 mM ammonium acetate in acetonitrile:isopropanol (4:3, *v*/*v*) containing 0.1% formic acid (solvent B). Both SLs and PLs were analyzed on a hybrid triple quadrupole/linear ion trap mass spectrometer (5500 QTRAP) equipped with an UHPLC system (CTC HTC PAL autosampler and Rheos Allegro pump or Shimadzu Nexera X2) using a multiple reaction monitoring based method in positive ion mode for sphingolipids and negative ion mode for molecular PLs. Identified lipids were quantified by normalizing against their respective internal standard [82] and total protein concentrations in the cell sample. The details for the procedure has been published before [83]. Total protein concentrations were determined using the Micro BCA™ Protein Assay Kit (Thermo Scientific Pierce Protein Research Products Cat. No. 23235, Rockford, IL, USA). Data processing was performed by MultiQuant, LipidView (AB Sciex) software and SAS.

### 4.5. miRNA Analysis

For quantification of RNA eluates, Nanodrop ND-1000 Instrument was used (Thermo Fisher Scientific, Darmstadt, Germany). Quality control was performed using Agilent 2100 Bioanalyzer and the Nano or Pico RNA Kit depending on Nanodrop RNA concentration (Pico Chip: 50–5000 pg/µL; Nano Chip: 5–500 ng/µL) according to the manufacturer’s instructions (Agilent Technologies, Santa Clara, CA, USA). The expression profiles of all miRBase release v21 human miRNAs were determined using Agilent Sureprint G3 Human miRNA (8 × 60 K) microarray slides. Each array targets 2549 microRNAs with 20 replicates per probe. RNA was labelled and hybridized using the Agilent’s miRNA Complete Labeling and Hybridization Kit according to manufacturer’s protocol. After hybridization for 20 h at 55 °C, the slides were washed twice and scanned using Agilent’s High Resolution Microarray Dx Scanner. Scan images were transformed to raw text data using Feature Extraction Software (Agilent Technologies, Santa Clara, CA, USA).

The signal to noise ratio (S/N) was set on 3 as a cut off value for the presence of miRNAs. To normalize the data across different arrays, quantile normalization [84] was applied using the robust multi-array average (RMA) algorithm [85]. All further analyses were based on these normalized and background subtracted data. Log2 fold change 1 was applied as a cut off value for determining the up- or downregulation of miRNAs in PHHs.

To find the expression patterns among miRNAs (presented in Figure 6), a complete linkage hierarchical clustering was applied using Euclidean distance. The patterns were based in relative expression to find similarities independent of the absolute expression intensity.

## 5. Conclusions

We identified alterations in the pathways of lipid and FA metabolism in the dedifferentiating PHHs, which may play fundamental roles in PHHs’ loss of phenotype in the culture. Our results suggest major modifications in the organization of the cellular membranes in the dedifferentiating PHHs, which may greatly impair their function and the way they store energy or secrete lipids. These alterations were concurrent with up- or downregulation of miRNAs with proven roles in the regulation of lipid or FA synthesis. However, the complete elucidation of the association between miRNAs and lipids during the dedifferentiating process remains to be further explored and validated.

## Figures and Tables

**Table 1:** List of key genes involved in the lipid and fatty acid metabolism and their function.

Genes	Functions	References
*CERS1*, *CERS2*, *CERS4*, *CERS5*, *CERS6*	Encoding enzymes that catalyze the synthesis of ceramides from sphingoid base and acyl-CoA substrates.	[33,44,45]
*ASAH1*, *ASAH2*	Encoding acid (*ASAH1*) and neutral (*ASAH2*) ceramidases that degrade ceramides to sphingosine and free fatty acids (FAs).	[29]
*SGMS1*, *SGMS2*	Synthesis of sphingomyelin from ceramides and crucial in ceramide and sphingomyelin homoeostasis.	[46]
*SMPD1*	Encoding a lysosomal acid sphingomyelinase hydrolyzing sphingomyelin to ceramide.	[47]
*UGCG*	Encoding the enzyme that catalyzes the first glycosylation step in glycosphingolipid biosynthesis.	[48]
*FADS1*, *FADS2*	Encoding for the enzymes inserting double bonds to pre-existing polyunsaturated fatty acid (PUFA) precursors. Critical for the synthesis and regulation of PUFAs.	[49]
*ELOVL1*, *ELOVL2*, *ELOVL3*, *ELOVL5*, *ELOVL6*, *ELOVL7*	Encoding enzymes that function in concert with FADSs and determine the rate of overall fatty acid elongation. *ELOVL1*, *ELOVL3*, *ELOVL6*, and *ELOVL7* are involved in elongation of saturated and monounsaturated FAs, while *ELOVL2* and *ELOVL5* are involved in elongation of PUFAs. *ELOVL5* also elongates some monounsaturated FAs, such as palmitoleic acid (FA 16:1).	[50]
*FASN*	Encoding the enzyme that determines the de-novo synthesis of FAs by synthesising palmitic acid (FA 16:0).	[40]
*SCD*	Encoding a rate-limiting enzyme in the cellular synthesis of monounsaturated FAs from saturated FAs.	[43]

## Supplementary Materials

Supplementary materials can be found at https://www.mdpi.com/1422-0067/20/12/2910/s1.

## Abbreviations

CE	cholesteryl ester
CerS	ceramide synthase
DAG	diacylglycerol
ELOVL	fatty acid elongase
ER	endoplasmic reticulum
FA	fatty acid
FADS	fatty acid desaturase
FASN	fatty acid synthase
Gb3	globotriaosylceramide
GCK	glucokiase
Glc/GalCer	glucosyl/galactosylceramide
GSL	glycosphingolipid
hLTR	human liver total RNA
HUFA	highly unsaturated fatty acid
LacCer	lactosylceramide
LCFA	long-chain fatty acid
miRNA	microRNA
MUFA	monounsaturated fatty acid
PC	phosphatidylcholine
PE	phosphatidylethanolamine
PCK1	phosphoenolpyruvate carboxykinase
PHH	primary human hepatocyte
PI	phosphatidylinositol
PKLR	liver-type pyruvate kinase
PL	phospholipid
PS	phosphatidylserine
PUFA	polyunsaturated fatty acid
qPCR	quantitative PCR
RXRα	retinoid X receptor alpha
SCD	stearoyl-CoA desaturase
SFA	saturated fatty acid
SL	sphingolipid
SM	sphingomyelin
SMS	sphingomyelin synthase
TAG	triacylglycerol
UGCG	UDP-glucose ceramide glucosyltransferase
VLCFA	very-long-chain fatty acid

## References

[1] Sahi J., Grepper S., Smith C. (2010). Hepatocytes as a tool in drug metabolism, transport and safety evaluations in drug discovery. Curr. Drug Discov. Technol..

[2] Kakisaka K., Cazanave S.C., Fingas C.D., Guicciardi M.E., Bronk S.F., Werneburg N.W., Mott J.L., Gores G.J. (2012). Mechanisms of lysophosphatidylcholine-induced hepatocyte lipoapoptosis. AJP Gastrointest. Liver Physiol..

[3] Ling J., Lewis J., Douglas D., Kneteman N.M., Vance D.E. (2013). Characterization of lipid and lipoprotein metabolism in primary human hepatocytes. Biochim. Biophys. Acta.

[4] Gordillo M., Evans T., Gouon-Evans V. (2015). Orchestrating liver development. Development.

[5] Schwartz R.E., Trehan K., Andrus L., Sheahan T.P., Ploss A., Duncan S.A., Rice C.M., Bhatia S.N. (2012). Modeling hepatitis C virus infection using human induced pluripotent stem cells. Proc. Natl. Acad. Sci. USA.

[6] Westerink W.M.A., Schoonen W.G.E.J. (2007). Cytochrome P450 enzyme levels in HepG2 cells and cryopreserved primary human hepatocytes and their induction in HepG2 cells. Toxicol. In Vitro.

[7] Michalopoulos G. (2007). Liver regeneration. J. Cell. Physiol..

[8] Elaut G., Henkens T., Papeleu P., Snykers S., Vinken M., Vanhaecke T., Rogiers V. (2006). Molecular mechanisms underlying the dedifferentiation process of isolated hepatocytes and their cultures. Curr. Drug Metab..

[9] Godoy P., Hewitt N.J., Albrecht U., Andersen M.E., Ansari N., Bhattacharya S., Bode J.G., Bolleyn J., Borner C., Böttger J. (2013). Recent advances in 2D and 3D in vitro systems using primary hepatocytes, alternative hepatocyte sources and non-parenchymal liver cells and their use in investigating mechanisms of hepatotoxicity, cell signaling and ADME. Arch. Toxicol..

[10] Godoy P., Hengstler J.G., Ilkavets I., Meyer C., Bachmann A., Müller A., Tuschl G., Mueller S.O., Dooley S. (2009). Extracellular matrix modulates sensitivity of hepatocytes to fibroblastoid dedifferentiation and transforming growth factor β-induced apoptosis. Hepatology.

[11] Gieseck R.L., Hannan N.R.F., Bort R., Hanley N.A., Drake R.A.L., Cameron G.W.W., Wynn T.A., Vallier L. (2014). Maturation of induced pluripotent stem cell derived hepatocytes by 3D-culture. PLoS ONE.

[12] Foster E., You J., Siltanen C., Patel D., Haque A., Anderson L., Revzin A. (2015). Heparin hydrogel sandwich cultures of primary hepatocytes. Eur. Polym. J..

[13] Heidariyan Z., Ghanian M.H., Ashjari M., Farzaneh Z., Najarasl M., Rezaei Larijani M., Piryaei A., Vosough M., Baharvand H. (2018). Efficient and cost-effective generation of hepatocyte-like cells through microparticle-mediated delivery of growth factors in a 3D culture of human pluripotent stem cells. Biomaterials.

[14] Zhong L., Gou J., Deng N., Shen H., He T., Zhang B.Q. (2015). Three-dimensional Co-culture of hepatic progenitor cells and mesenchymal stem cells in vitro and in vivo. Microsc. Res. Tech..

[15] Kruitwagen H.S., Oosterhoff L.A., Vernooij I.G.W.H., Schrall I.M., van Wolferen M.E., Bannink F., Roesch C., van Uden L., Molenaar M.R., Helms J.B. (2017). Long-term adult feline liver organoid cultures for disease modeling of hepatic steatosis. Stem Cell Rep..

[16] Gijbels E., Vanhaecke T., Vinken M. (2019). Establishment of sandwich cultures of primary human hepatocytes. Methods in Molecular Biology.

[17] Beigel J., Fella K., Kramer P.J., Kroeger M., Hewitt P. (2008). Genomics and proteomics analysis of cultured primary rat hepatocytes. Toxicol. In Vitro.

[18] Heslop J.A., Rowe C., Walsh J., Sison-Young R., Jenkins R., Kamalian L., Kia R., Hay D., Jones R.P., Malik H.Z. (2017). Mechanistic evaluation of primary human hepatocyte culture using global proteomic analysis reveals a selective dedifferentiation profile. Arch. Toxicol..

[19] Kim Y., Lasher C.D., Milford L.M., Murali T.M., Rajagopalan P. (2010). A comparative study of genome-wide transcriptional profiles of primary hepatocytes in collagen sandwich and monolayer cultures. Tissue Eng. Part C. Methods.

[20] Lasher C.D., Rajagopalan P., Murali T.M. (2011). Discovering networks of perturbed biological processes in hepatocyte cultures. PLoS ONE.

[21] Rowe C., Goldring C.E.P., Kitteringham N.R., Jenkins R.E., Lane B.S., Sanderson C., Elliott V., Platt V., Metcalfe P., Park B.K. (2010). Network analysis of primary hepatocyte dedifferentiation using a shotgun proteomics approach. J. Proteome Res..

[22] Lauschke V.M., Vorrink S.U., Moro S.M.L., Rezayee F., Nordling Å., Hendriks D.F.G., Bell C.C., Sison-Young R., Park B.K., Goldring C.E. (2016). Massive rearrangements of cellular MicroRNA signatures are key drivers of hepatocyte dedifferentiation. Hepatology.

[23] Muro E., Atilla-Gokcumen G.E., Eggert U.S. (2014). Lipids in cell biology: How can we understand them better?. Mol. Biol. Cell.

[24] Zanger U.M., Schwab M. (2013). Cytochrome P450 enzymes in drug metabolism: Regulation of gene expression, enzyme activities, and impact of genetic variation. Pharmacol. Ther..

[25] Cai S.-Y., He H., Nguyen T., Mennone A., Boyer J.L. (2010). Retinoic acid represses CYP7A1 expression in human hepatocytes and HepG2 cells by FXR/RXR-dependent and independent mechanisms. J. Lipid Res..

[26] Lewiń-ska M., Zelenko U., Merzel F., Grdadolnik S.G., Murray J.C., Rozman D. (2013). Polymorphisms of CYP51A1 from cholesterol synthesis: Associations with birth weight and maternal lipid levels and impact on CYP51 protein structure. PLoS ONE.

[27] Kiamehr M. (2019). Induced pluripotent stem cell-derived hepatocyte-like cells; the lipid status in differentiation, functionality, and de-differentiation of hepatic cells. Ph.D. Dissertation.

[28] Baker T.K., Carfagna M.A., Gao H., Dow E.R., Li Q., Searfoss G.H., Ryan T.P. (2001). Temporal gene expression analysis of monolayer cultured rat hepatocytes. Chem. Res. Toxicol..

[29] Gault C.R., Obeid L.M., Hannun Y.A. (2010). An overview of sphingolipid metabolism: From synthesis to breakdown. Adv. Exp. Med. Biol..

[30] Bartke N., Hannun Y.A. (2008). Bioactive sphingolipids: Metabolism and function. J. Lipid Res..

[31] Delgado A., Casas J., Llebaria A., Abad J.L., Fabrias G. (2006). Inhibitors of sphingolipid metabolism enzymes. Biochim. Biophys. Acta - Biomembr..

[32] Jennemann R., Gröne H.J. (2013). Cell-specific in vivo functions of glycosphingolipids: Lessons from genetic deletions of enzymes involved in glycosphingolipid synthesis. Prog. Lipid Res..

[33] Cingolani F., Futerman A.H., Casas J. (2016). Ceramide synthases in biomedical research. Chem. Phys. Lipids.

[34] Saddoughi S.A., Song P., Ogretmen B. (2008). Roles of Bioactive Sphingolipids in Cancer Biology and Therapeutics. Lipids Health Dis..

[35] Mesicek J., Lee H., Feldman T., Jiang X., Skobeleva A., Berdyshev E.V., Haimovitz-Friedman A., Fuks Z., Kolesnick R. (2010). Ceramide synthases 2, 5, and 6 confer distinct roles in radiation-induced apoptosis in HeLa cells. Cell. Signal..

[36] Hannun Y.A., Luberto C. (2004). Lipid metabolism: Ceramide transfer protein adds a new dimension. Curr. Biol..

[37] Li Z., Hailemariam T.K., Zhou H., Li Y., Duckworth D.C., Peake D.A., Zhang Y., Kuo M.S., Cao G., Jiang X.C. (2007). Inhibition of sphingomyelin synthase (SMS) affects intracellular sphingomyelin accumulation and plasma membrane lipid organization. Biochim. Biophys. Acta-Mol. Cell Biol. Lipids.

[38] Simons K., Gerl M.J. (2010). Revitalizing membrane rafts: New tools and insights. Nat. Rev. Mol. Cell Biol..

[39] Ishibashi Y., Kohyama-Koganeya A., Hirabayashi Y. (2013). New insights on glucosylated lipids: Metabolism and functions. Biochim. Biophys. Acta-Mol. Cell Biol. Lipids.

[40] Jensen-Urstad A.P.L., Semenkovich C.F. (2012). Fatty acid synthase and liver triglyceride metabolism: Housekeeper or messenger?. Biochim. Biophys. Acta-Mol. Cell Biol. Lipids.

[41] Ntambi J.M., Miyazaki M. (2004). Regulation of stearoyl-CoA desaturases and role in metabolism. Prog. Lipid Res..

[42] Jakobsson A., Westerberg R., Jacobsson A. (2006). Fatty acid elongases in mammals: Their regulation and roles in metabolism. Prog. Lipid Res..

[43] Ntambi J.M. (1999). Regulation of stearoyl-CoA desaturase by polyunsaturated fatty acids and cholesterol. J. Lipid Res..

[44] Park J.W., Park W.J., Futerman A.H. (2014). Ceramide synthases as potential targets for therapeutic intervention in human diseases. Biochim. Biophys. Acta-Mol. Cell Biol. Lipids.

[45] Mullen T.D., Hannun Y.A., Obeid L.M. (2012). Ceramide synthases at the centre of sphingolipid metabolism and biology. Biochem. J..

[46] Adada M., Luberto C., Canals D. (2016). Inhibitors of the sphingomyelin cycle: Sphingomyelin synthases and sphingomyelinases. Chem. Phys. Lipids.

[47] Kim W.J., Okimoto R.A., Purton L.E., Goodwin M., Haserlat S.M., Dayyan F., Sweetser D.A., Mcclatchey A.I., Bernard O.A., Look A.T. (2008). Mutations in the neutral sphingomyelinase gene Smpd3 implicate the ceramide pathway in human leukemias. Blood.

[48] D’Angelo G., Capasso S., Sticco L., Russo D. (2013). Glycosphingolipids: Synthesis and functions. FEBS J..

[49] Lee J.M., Lee H., Kang S.B., Park W.J. (2016). Fatty acid desaturases, polyunsaturated fatty acid regulation, and biotechnological advances. Nutrients.

[50] Jump D.B. (2009). Mammalian fatty acid elongases. Methods Mol. Biol..

[51] Duplus E., Forest C. (2002). Is there a single mechanism for fatty acid regulation of gene transcription?. Biochem. Pharmacol..

[52] Currie E., Schulze A., Zechner R., Walther T.C., Farese R.V. (2013). Cellular fatty acid metabolism and cancer. Cell Metab..

[53] Finelli C., Tarantino G. (2012). Have guidelines addressing physical activity been established in nonalcoholic fatty liver disease?. World J. Gastroenterol..

[54] Fraczek J., Bolleyn J., Vanhaecke T., Rogiers V., Vinken M. (2013). Primary hepatocyte cultures for pharmaco-toxicological studies: At the busy crossroad of various anti-dedifferentiation strategies. Arch. Toxicol..

[55] Yang Z., Cappello T., Wang L. (2015). Emerging role of microRNAs in lipid metabolism. Acta Pharm. Sin. B.

[56] Zhang M., Sun W., Zhou M., Tang Y. (2017). MicroRNA-27a regulates hepatic lipid metabolism and alleviates NAFLD via repressing FAS and SCD1. Sci. Rep..

[57] Novák J., Olejníčková V., Tkáčová N., Santulli G., Santulli G. (2015). Mechanistic role of microRNAs in coupling lipid metabolism and atherosclerosis. Advances in Experimental Medicine and Biology.

[58] Takagi S., Nakajima M., Kida K., Yamaura Y., Fukami T., Yokoi T. (2010). MicroRNAs regulate human hepatocyte nuclear factor 4alpha, modulating the expression of metabolic enzymes and cell cycle. J. Biol. Chem..

[59] Loyer X., Paradis V., Hénique C., Vion A.C., Colnot N., Guerin C.L., Devue C., On S., Scetbun J., Romain M. (2016). Liver microRNA-21 is overexpressed in non-alcoholic steatohepatitis and contributes to the disease in experimental models by inhibiting PPARα expression. Gut.

[60] Goodwin B., Jones S.A., Price R.R., Watson M.A., McKee D.D., Moore L.B., Galardi C., Wilson J.G., Lewis M.C., Roth M.E. (2000). A regulatory cascade of the nuclear receptors FXR, SHP-1, and LRH-1 represses bile acid biosynthesis. Mol. Cell.

[61] Oda Y., Nakajima M., Tsuneyama K., Takamiya M., Aoki Y., Fukami T., Yokoi T. (2014). Retinoid X receptor α in human liver is regulated by miR-34a. Biochem. Pharmacol..

[62] Rodgers J.T., Puigserver P. (2007). Fasting-dependent glucose and lipid metabolic response through hepatic sirtuin 1. Proc. Natl. Acad. Sci. USA.

[63] Kim H.R., Roe J.S., Lee J.E., Cho E.J., Youn H.D. (2013). P53 regulates glucose metabolism by miR-34a. Biochem. Biophys. Res. Commun..

[64] Ye X., Li M., Hou T., Gao T., Zhu W., Yang Y. (2017). Sirtuins in glucose and lipid metabolism. Oncotarget.

[65] Reyes R.K., Motiwala T., Jacob S.T. (2014). Regulation of glucose metabolism in hepatocarcinogenesis by MicroRNAs. Gene Expr..

[66] Wang B., Hsu S.H., Frankel W., Ghoshal K., Jacob S.T. (2012). Stat3-mediated activation of microRNA-23a suppresses gluconeogenesis in hepatocellular carcinoma by down-regulating Glucose-6-phosphatase and peroxisome proliferator-activated receptor gamma, coactivator 1 alpha. Hepatology.

[67] Ji J., Zhang J., Huang G., Qian J., Wang X., Mei S. (2009). Over-expressed microRNA-27a and 27b influence fat accumulation and cell proliferation during rat hepatic stellate cell activation. FEBS Lett..

[68] Shirasaki T., Honda M., Shimakami T., Horii R., Yamashita T., Sakai Y., Sakai A., Okada H., Watanabe R., Murakami S. (2013). MicroRNA-27a regulates lipid metabolism and inhibits hepatitis C virus replication in human hepatoma cells. J. Virol..

[69] Ahn J., Lee H., Jung C.H., Ha T. (2012). Lycopene inhibits hepatic steatosis via microRNA-21-induced downregulation of fatty acid-binding protein 7 in mice fed a high-fat diet. Mol. Nutr. Food Res..

[70] Kida K., Nakajima M., Mohri T., Oda Y., Takagi S., Fukami T., Yokoi T. (2011). PPARα is regulated by miR-21 and miR-27b in human liver. Pharm. Res..

[71] Fernández-Alvarez A., Soledad Alvarez M., Gonzalez R., Cucarella C., Muntané J., Casado M. (2011). Human SREBP1c expression in liver is directly regulated by Peroxisome Proliferator-activated Receptor α (PPARα). J. Biol. Chem..

[72] Fan J., Li H., Nie X., Yin Z., Zhao Y., Chen C., Wen Wang D. (2017). MiR-30c-5p ameliorates hepatic steatosis in leptin receptor-deficient (db/db) mice via down-regulating FASN. Oncotarget.

[73] Irani S., Hussain M.M. (2015). Role of microRNA-30c in lipid metabolism, adipogenesis, cardiac remodeling and cancer. Curr. Opin. Lipidol..

[74] Esau C., Davis S., Murray S.F., Yu X.X., Pandey S.K., Pear M., Watts L., Booten S.L., Graham M., McKay R. (2006). miR-122 regulation of lipid metabolism revealed by in vivo antisense targeting. Cell Metab..

[75] Poy M.N., Spranger M., Stoffel M. (2007). microRNAs and the regulation of glucose and lipid metabolism. Diabetes, Obes. Metab..

[76] Ståhlman M., Ejsing C.S., Tarasov K., Perman J., Borén J., Ekroos K. (2009). High-throughput shotgun lipidomics by quadrupole time-of-flight mass spectrometry. J. Chromatogr. B Anal. Technol. Biomed. Life Sci..

[77] Kiamehr M., Viiri L.E., Vihervaara T., Koistinen K.M., Hilvo M., Ekroos K., Kakela R., Aalto-Setala K. (2017). Lipidomic profiling of patient-specific iPSC-derived hepatocyte-like cells. DMM Dis. Model. Mech..

[78] Heiskanen L.A., Suoniemi M., Ta H.X., Tarasov K., Ekroos K. (2013). Long-Term Performance and Stability of Molecular Shotgun Lipidomic Analysis of Human Plasma Samples. Anal. Chem..

[79] Ekroos K., Ejsing C.S., Bahr U., Karas M., Simons K., Shevchenko A. (2003). Charting molecular composition of phosphatidylcholines by fatty acid scanning and ion trap MS3 fragmentation. J. Lipid Res..

[80] Ekroos K., Chernushevich I.V., Simons K., Shevchenko A. (2002). Quantitative profiling of phospholipids by multiple precursor ion scanning on a hybrid quadrupole time-of-flight mass spectrometer. Anal. Chem..

[81] Merrill A.H., Sullards M.C., Allegood J.C., Kelly S., Wang E. (2005). Sphingolipidomics: High-throughput, structure-specific, and quantitative analysis of sphingolipids by liquid chromatography tandem mass spectrometry. Methods.

[82] Ejsing C.S., Duchoslav E., Sampaio J., Simons K., Bonner R., Thiele C., Ekroos K., Shevchenko A. (2006). Automated identification and quantification of glycerophospholipid molecular species by multiple precursor ion scanning. Anal. Chem..

[83] Kiamehr M., Alexanova A., Viiri L.E., Heiskanen L., Vihervaara T., Kauhanen D., Ekroos K., Laaksonen R., Käkelä R., Aalto-Setälä K. (2019). hiPSC-derived hepatocytes closely mimic the lipid profile of primary hepatocytes: A future personalised cell model for studying the lipid metabolism of the liver. J. Cell. Physiol..

[84] Bolstad B.M., Irizarry R.A., Astrand M., Speed T.P. (2003). A comparison of normalization methods for high density oligonucleotide array data based on variance and bias. Bioinformatics.

[85] Irizarry R.A., Bravo H.C., Irizarry R.A., Irizarry R., Hobbs B., Collin F., Beazer-Barclay Y., Antonellis K., Scherf U., Speed T. (2003). Exploration, normalization, and summaries of high density oligonucleotide array probe level data. Biostatistics.

