# Supplementary material for: Dedifferentiation of Primary Hepatocytes is Accompanied with Reorganization of Lipid Metabolism Indicated by Altered Molecular Lipid and miRNA Profiles
Source: Int J Mol Sci. 2019 Jun 14;20(12):2910. doi: 10.3390/ijms20122910 (PMC6627955; doi:10.3390/ijms20122910)
Supplement: Supplementary file 1 [file ijms-20-02910-s001.zip › so proof done/Table S1_RT-qPCR.pdf]

Supplemental table S1a. List of the studied liver-specific genes in PHHs using SYBR green_based real-time quantitative PCR (QPCR). GAPDH was used as the endogenous control gene.

Gene symbol	Gene name	Forward primer 5'-3'	Reverse primer 5'-3'
FOXA2	Forkhead box A2	AAGACCTACAGGCGCAGCT	CATCTTGTTGGGGCTCTGC
AFP	Alpha fetoprotein	CGCTGCAAACGATGAAGCAG	AATCTGCAATGACAGCCTCAAG
ALB	Albumin	GAAAAGTGGGCAGCAAATGT	GGTTCAGGACCACGGATAGA
GAPDH	Glyceraldehyde-3-phosphate dehydrogenase	AGCCACATCGCTCAGACACC	GTACTCAGCGCCAGCATCG

Supplemental table S1b. List of the studied genes related to the lipids, fatty acids, as well as glucose metabolism in cultured primary human hepatocytes using TaqMan_based real-time quantitative PCR (qPCR) assays. Both GAPDH and B2M were used as the endogenous controls.

Gene symbol	Gene name	TaqMan assay ID
FASN	Fatty acid synthase	Hs01005622_m1
SCD	Stearoyl-CoA desaturase	Hs01682761_m1
FADS1	Fatty Acid Desaturase 1	Hs01096545_m1
FADS2	Fatty Acid Desaturase 2	Hs00927433_m1
ELOVL1	Elongation of very-long chain fatty acids 1	Hs00967951_g1
ELOVL2	Elongation of very-long chain fatty acids 2	Hs00214936_m1
ELOVL3	Elongation of very-long chain fatty acids 3	Hs00537016_m1
ELOVL5	Elongation of very-long chain fatty acids 5	Hs01094711_m1
ELOVL6	Elongation of very-long chain fatty acids 6	Hs00907565_m1
ELOVL7	Elongation of very-long chain fatty acids 7	HS00405151_m1
GCK	Glucokinase	Hs01564555_m1
PKLR	Liver-type pyruvate kinase	Hs00176075_m1
PCK1	Phosphoenolpyruvate carboxykinase	Hs00159918_m1
CERS1	Ceramide synthase 1	Hs04195319_s1
CERS2	Ceramide synthase 2	Hs00371958_g1
CERS4	Ceramide synthase 4	Hs00226114_m1
CERS5	Ceramide synthase 5	Hs00908759_m1
CERS6	Ceramide synthase 6	Hs00826756_m1
SGMS1	Sphingomyelin synthase 1	Hs00983630_m1
SGMS2	Sphingomyelin synthase 2	Hs00380453_m1
UGCG	UDP-glucose ceramide glucosyltransferase	Hs00234293_m1
SMPD1	Sphingomyelin phosphodiesterase 1	Hs03679347_g1
ASAH1	N-acylsphingosine amidohydrolase (acid ceramidase) 1	Hs00602774_m1
ASAH2	N-acylsphingosine amidohydrolase (non-lysosomal ceramidase) 2	Hs01025305_m1
B2M	Beta-2-microglobulin	Hs99999907_m1
GAPDH	Glyceraldehyde-3-phosphate dehydrogenase	Hs99999905_m1
